# *catena*-Poly[[chloridomercury(II)]-μ-1,4-diaza­bicyclo­[2.2.2]octane-κ^2^ *N*:*N*′-[chlorido­mercury(II)]-di-μ-chlorido]

**DOI:** 10.1107/S1600536809043839

**Published:** 2009-10-31

**Authors:** Shao-Ming Fang, Min Hu, Song-Tao Ma, Chun-Sen Liu

**Affiliations:** a Zhengzhou University of Light Industry, Henan Provincial Key Laboratory of Surface & Interface Science, Henan, Zhengzhou 450002, People’s Republic of China

## Abstract

In the title coordination polymer, [Hg_2_Cl_4_(C_6_H_12_N_2_)]_*n*_, each Hg^II^ center within the chain is four-coordinated by one terminal Cl atom, two bridging μ_2_-Cl atoms, and one N-atom donor from a μ_2_-1,4-diaza­bicyclo­[2.2.2]octane (μ_2_-daco) ligand in a distorted tetra­hedral geometry. The daco ligand acts as an end-to-end bridging ligand and bridges adjacent Hg^II^ centers, forming a chain running along [001]. Weak C—H⋯Cl hydrogen-bonding inter­actions link the chains into a three-dimensional network. Comparison of the structural differences with previous findings suggests that the space between the two N donors, as well as the skeletal rigidity in *N*-heterocyclic linear ligands, may play an important role in the construction of such supra­molecular networks.

## Related literature

For a related structure, see: Pickardt *et al.* (1995 ▶). For functional materials, see: Chen, Kang & Su (2006 ▶); Fang *et al.* (2009 ▶); Liu *et al.* (2007 ▶); Ma *et al.* (2009 ▶); Tranchemontagne *et al.* (2009 ▶); Uemura *et al.* (2009 ▶); Xue *et al.* (2008 ▶). For N-containing hetercyclic bridging ligands, see: Batten *et al.* (2002 ▶); Chen *et al.* (2007 ▶); Culp *et al.* (2008 ▶); Kaim (1983 ▶); Leininger *et al.* (2000 ▶); Richardson & Steel (2003 ▶); Steel (2005 ▶). For 4,4′-bipyridine and pyrazine extended assemblies, see: Arpi *et al.* (2006 ▶); Chen, Wang *et al.* (2006 ▶); Choi *et al.* (2009 ▶); Derossi *et al.* (2007 ▶); Du *et al.* (2007 ▶); Liu *et al.* (2006 ▶); Li *et al.*(2008 ▶); Ramírez *et al.* (2009 ▶); Qiu *et al.* (2008 ▶); Nockemann & Meyer (2004 ▶); Xie & Wu (2007 ▶). For daco complexes, see: Dybtsev *et al.* (2004 ▶); Li *et al.* (2006 ▶); Rao & Rao (2007 ▶); Steel (2005 ▶). For factors determining the crystal packing, see: Kitagawa *et al.* (2004 ▶). For Hg—N and Hg—Cl bond distances and bond angles about Hg, see: Orpen *et al.* (1989 ▶); Wang *et al.* (2007 ▶).
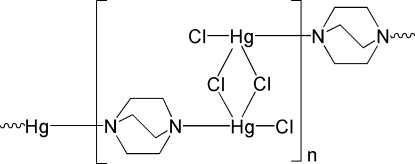


## Experimental

### 

#### Crystal data

[Hg_2_Cl_4_(C_6_H_12_N_2_)]
*M* *_r_* = 327.58
Orthorhombic, 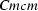

*a* = 9.2518 (9) Å
*b* = 8.8244 (9) Å
*c* = 14.7531 (8) Å
*V* = 1204.47 (18) Å^3^
*Z* = 8
Mo *K*α radiation
μ = 26.31 mm^−1^
*T* = 293 K
0.05 × 0.04 × 0.02 mm

#### Data collection

Bruker SMART CCD area-detector diffractometer
Absorption correction: multi-scan (*SADABS*; Sheldrick, 1996 ▶) *T*_min_ = 0.353, *T*_max_ = 0.621
1322 measured reflections
586 independent reflections
392 reflections with *I* > 2σ(*I*)
*R*_int_ = 0.040

#### Refinement

*R*[*F* ^2^ > 2σ(*F* ^2^)] = 0.037
*wR*(*F* ^2^) = 0.047
*S* = 0.96
586 reflections
39 parameters
6 restraints
H-atom parameters constrained
Δρ_max_ = 1.41 e Å^−3^
Δρ_min_ = −1.59 e Å^−3^

### 

Data collection: *SMART* (Bruker, 2007 ▶); cell refinement: *SAINT* (Bruker, 2007 ▶); data reduction: *SAINT*; program(s) used to solve structure: *SHELXS97* (Sheldrick, 2008 ▶); program(s) used to refine structure: *SHELXL97* (Sheldrick, 2008 ▶); molecular graphics: *SHELXTL* (Sheldrick, 2008 ▶); software used to prepare material for publication: *SHELXTL* and *PLATON* (Spek, 2009 ▶).

## Supplementary Material

Crystal structure: contains datablocks I, global. DOI: 10.1107/S1600536809043839/su2148sup1.cif

Structure factors: contains datablocks I. DOI: 10.1107/S1600536809043839/su2148Isup2.hkl

Additional supplementary materials:  crystallographic information; 3D view; checkCIF report

## Figures and Tables

**Table 1:** Hydrogen-bond geometry (Å, °)

*D*—H⋯*A*	*D*—H	H⋯*A*	*D*⋯*A*	*D*—H⋯*A*
C2—H2*A*⋯Cl2^i^	0.97	2.77	3.708 (8)	163
C2—H2*B*⋯Cl2^ii^	0.97	2.78	3.678 (8)	154

Symmetry codes: (i) 
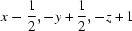
; (ii) 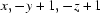.

**Fig. 1.**
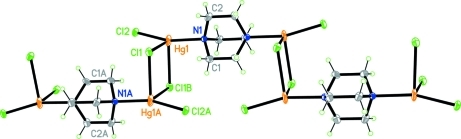
The one-dimensional molecular structure of the title complex, propagating in the [001] direction. Displacement ellipsoids are drawn at the 30% probability level. The atoms labeled with the suffixes A and B are generated by the symmetry operations (x, -y, -z + 1; -x + 1, -y, -z + 1), respectively.

**Fig. 2.**
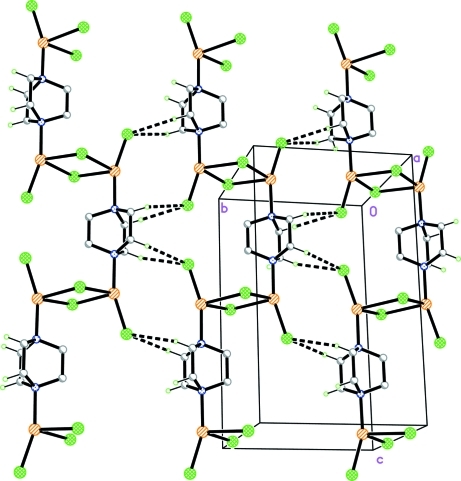
The two-dimensional net, viewed along the a axis, formed by the interchain C–H···Cl hydrogen-bonds (dashed lines). For clarity, only H-atoms involved in hydrogen-bonding are shown.

**Fig. 3.**
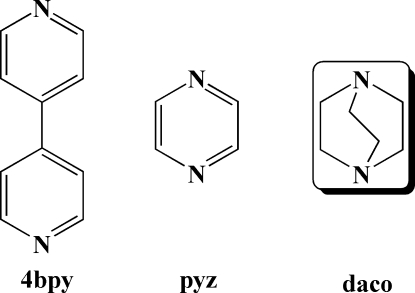
Structures of 4bpy, pyz and daco.

**Fig. 4.**
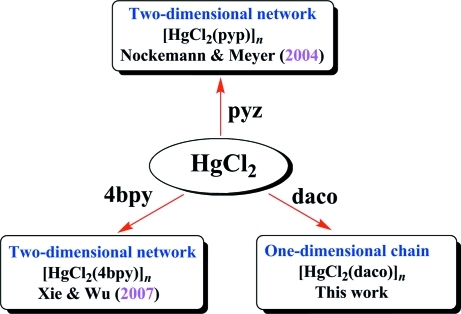
Reaction scheme.

## supplementary crystallographic information

### Comment

The rational engineering and controlled preparation of novel coordination complexes are currently of great interest in coordination and supramolecular chemistry not only because of their intriguing structural diversities but also their potential applications as functional materials (Chen, Kang & Su, 2006; Fang *et al.*, 2009; Liu *et al.*, 2007; Ma *et al.*, 2009; Tranchemontagne *et al.*, 2009; Uemura *et al.*, 2009; Xue *et al.*, 2008). One of the most successful strategies for constructing such complexes has been the assembly reaction of different metal ions (as nodes) with well designed organic ligands (as building blocks), which, so far, has been at an evolutionary stage with the current focus mainly on understanding the factors to determine the crystal packing as well as exploring relevant potential properties (Kitagawa *et al.*, 2004). N-containing heterocyclic ligands are extensively used as bridging ligands in coordination and metallosupramolecular chemistry (Batten *et al.*, 2002; Chen *et al.*, 2007; Culp *et al.*, 2008; Kaim, 1983; Leininger *et al.*, 2000; Richardson & Steel, 2003; Steel, 2005). For examples, the use of 4,4'-bipyridine (4bpy) and pyrazine (pyz) has resulted in a large number of extended assemblies including helical networks as well as diamondoid, honeycomb, square-grid, ribbon, grid, T-shaped, Ladder, brick wall, and octahedral frameworks (Arpi *et al.*, 2006; Chen, Wang *et al.*, 2006; Choi *et al.*, 2009; Derossi *et al.*, 2007; Du *et al.*, 2007; Liu *et al.*, 2006; Li *et al.*, 2008; Ramírez *et al.*, 2009; Qiu *et al.*, 2008).

In comparison with 4bpy and pyz ligands (Fig. 3), however, 1,4-diazabicyclo[2.2.2]octane (daco) acts as a flexible N-containing heterocyclic bridging ligand with a skeletal separation of *ca*6 Å between two N donors, shorter than that of *ca* 7 and 11 Å for 4bpy and pyz, respectively (Dybtsev *et al.*, 2004, Li *et al.*, 2006; Rao & Rao, 2007; Steel, 2005). As part of a study on the effect of the space between two N donors or the skeletal rigidity in linear N-containing heterocyclic ligands on the self-assembly of coordination complexes, we have chosen to use 1,4-diazabicyclo[2.2.2]octane (daco) instead of 4bpy or pyz to construct the title compound, a new one-dimensional Hg^II^ coordination polymer.

To explore the coordination possibility of a series of N-containing heterocyclic linear ligands, Xie & Wu (2007) as well as Nockemann & Meyer (2004) have selected 4,4'-bipyridine (4bpy) and pyrazine (pyz) to react with Hg^II ^atom, obtaining two different two-dimensional neutral networks, [HgCl_2_(4bpy)]_n_ (*A*) and [HgCl_2_(pyz)]_n _(*B*), respectively (Fig. 4). In these two structures, the Hg atoms are both in octahedral coordination environments, coordinated by four µ_2_-Cl atoms and two µ_2_-4bpy for *A* as well as four µ_2_-Cl atoms and two µ_2_-pyz for *B* in *trans* positions. The relevant one-dimensional chain motifs bridged by µ_2_-4bpy or µ_2_-pyz in complexes *A* and *B *are further interlinked to form the two-dimensional layers *via* the µ_2_-Cl atoms. In this contribution, however, when we used HgCl_2_ to react with 1,4-diazabicyclo[2.2.2] octane (daco) under a conventional solution diffusion condition, the title one-dimensional coordination polymer, [Hg_2_Cl_4_(daco)]_n_, was produced.

The crystal structure of the title compound consists of one-dimensional (1D) neutral [Hg_2_Cl_4_(daco)]_n_ chains (Fig. 1). The Hg^II ^center is four-coordinated, by three Cl atoms (one terminal Cl and two µ_2_-Cl atoms) and one N-atom donor from one daco ligand, with a distorted tetrahedral geometry. Two µ_2_-Cl atoms bridge adjacent Hg^II ^centers to form one planar four-membered rings composed of Hg1–Cl1–Hg1^i^–Cl1^ii^ with a non-bonding Hg(1)···Hg(1^i^) separation of 4.067 (1) Å [symmetry codes (i) = *x*, *–y*, 1–z; (ii) = 1–*x*, *–y*, 1–z]. Each daco ligand takes a µ_2_-bridging coordination mode to connect the adjacent Hg^II^centers, generating a one-dimensional chain running along the [001] direction. The Hg–N and Hg–Cl bond distances as well as the bond angles around each Hg^II^ center are within the expected range for such complexes (Orpen *et al.*, 1989; Wang *et al.*, 2007). Moreover, adjacent 1D [Hg_2_Cl_4_(daco)]_n_ chains are arranged into two-dimensional layers, running parallel to the (100) plane, by interchain C–H···Cl hydrogen-bonding interactions between the daco ligands and the terminal Cl atoms (Fig. 2 and Table 1).

Thus, in comparison with the previous findings, the present work reveals that the space between two N donors, or the skeletal rigidity of N-containing heterocyclic linear ligands, could play an important role in the final structure of the relate coordination complexes. This fact may offer the means to construct new coordination architectures with potentially useful properties by varying of the space between the two N donors, or the rigidity of skeleton of N-containing heterocyclic ligands. It is noteworthy that mercury(II) metal halide-daco materials are relatively rare, and to the best of our knowledge, only one halide-daco complex has been reported previously, *viz*. HgI_2_-daco (Pickardt *et al.*, 1995). This complex, [HgI_2_(daco)]_n_, also exhibits a zigzag one-dimensional structure, but here the I-atoms are all terminally coordinated to the metal centers, while in the title complex one of the Cl atoms act as a bridging atom.

### Experimental

A solution of 1,4-Diazabicyclo[2.2.2]octane (daco) (0.05 mmol) in CH_3_OH (10 ml) was carefully layered on top of an aqueous solution (15 ml) of HgCl_2_ (0.1 mmol) in a test tube. Colorless single crystals suitable for X-ray analysis appeared at the tube wall after *ca* one month at room temperature (yield ~30% based on daco). Elemental analysis calculated for (C_3_H_6_Cl_2_HgN): H 1.85, C 11.00, N 4.28%; found: H 1.78, C 10.87, N 4.24%.

### Refinement

H atoms were included in calculated positions and treated as riding atoms: C—H = 0.97 Å, with *U*_iso_(H) = 1.2 *U*_eq_(C).

### Crystal data

**Table:**

[Hg_2_Cl_4_(C_6_H_12_N_2_)]	*F*(000) = 1160
*M**_r_* = 327.58	*D*_x_ = 3.613 Mg m^−^^3^
Orthorhombic, *C**m**c**m*	Mo *K*α radiation, λ = 0.71073 Å
Hall symbol: -C 2c 2	Cell parameters from 453 reflections
*a* = 9.2518 (9) Å	θ = 3.2–30.3°
*b* = 8.8244 (9) Å	µ = 26.31 mm^−^^1^
*c* = 14.7531 (8) Å	*T* = 293 K
*V* = 1204.47 (18) Å^3^	Block, colorless
*Z* = 8	0.05 × 0.04 × 0.02 mm

### Data collection

**Table:**

Bruker SMART CCD area-detector diffractometer	586 independent reflections
Radiation source: fine-focus sealed tube	392 reflections with *I* > 2σ(*I*)
graphite	*R*_int_ = 0.040
φ and ω scans	θ_max_ = 25.0°, θ_min_ = 3.2°
Absorption correction: multi-scan (*SADABS*; Sheldrick, 1996)	*h* = −10→10
*T*_min_ = 0.353, *T*_max_ = 0.621	*k* = −10→8
1322 measured reflections	*l* = −17→9

### Refinement

**Table:**

Refinement on *F*^2^	Primary atom site location: structure-invariant direct methods
Least-squares matrix: full	Secondary atom site location: difference Fourier map
*R*[*F*^2^ > 2σ(*F*^2^)] = 0.037	Hydrogen site location: inferred from neighbouring sites
*wR*(*F*^2^) = 0.047	H-atom parameters constrained
*S* = 0.96	*w* = 1/[σ^2^(*F*_o_^2^) + (0.0085*P*)^2^] where *P* = (*F*_o_^2^ + 2*F*_c_^2^)/3
586 reflections	(Δ/σ)_max_ < 0.001
39 parameters	Δρ_max_ = 1.41 e Å^−^^3^
6 restraints	Δρ_min_ = −1.59 e Å^−^^3^

### Special details

**Table:**

Geometry. All e.s.d.'s (except the e.s.d. in the dihedral angle between two l.s. planes) are estimated using the full covariance matrix. The cell e.s.d.'s are taken into account individually in the estimation of e.s.d.'s in distances, angles and torsion angles; correlations between e.s.d.'s in cell parameters are only used when they are defined by crystal symmetry. An approximate (isotropic) treatment of cell e.s.d.'s is used for estimating e.s.d.'s involving l.s. planes.
Refinement. Refinement of *F*^2^ against ALL reflections. The weighted *R*-factor *wR* and goodness of fit *S* are based on *F*^2^, conventional *R*-factors *R* are based on *F*, with *F* set to zero for negative *F*^2^. The threshold expression of *F*^2^ > σ(*F*^2^) is used only for calculating *R*-factors(gt) *etc*. and is not relevant to the choice of reflections for refinement. *R*-factors based on *F*^2^ are statistically about twice as large as those based on *F*, and *R*- factors based on ALL data will be even larger.

### Fractional atomic coordinates and isotropic or equivalent isotropic displacement parameters (Å^2^)

**Table:**

	*x*	*y*	*z*	*U*_iso_*/*U*_eq_	Occ. (<1)
Hg1	0.5000	0.22915 (6)	0.51455 (3)	0.0533 (2)	
C1	0.5000	0.0735 (12)	0.6983 (7)	0.036 (3)	
H1A	0.5849	0.0205	0.6763	0.043*	0.50
H1B	0.4151	0.0205	0.6763	0.043*	0.50
C2	0.3708 (8)	0.3114 (9)	0.6975 (5)	0.032 (2)	
H2A	0.2843	0.2615	0.6754	0.039*	
H2B	0.3704	0.4150	0.6754	0.039*	
Cl1	0.2906 (3)	0.0000	0.5000	0.0344 (8)	
Cl2	0.5000	0.3087 (3)	0.3653 (2)	0.0349 (9)	
N1	0.5000	0.2318 (11)	0.6631 (6)	0.022 (2)	

### Atomic displacement parameters (Å^2^)

**Table:**

	*U*^11^	*U*^22^	*U*^33^	*U*^12^	*U*^13^	*U*^23^
Hg1	0.0692 (4)	0.0785 (5)	0.0122 (3)	0.000	0.000	0.0076 (3)
C1	0.055 (9)	0.017 (6)	0.036 (8)	0.000	0.000	0.006 (5)
C2	0.016 (5)	0.050 (6)	0.031 (5)	−0.003 (4)	−0.003 (4)	0.008 (4)
Cl1	0.0244 (17)	0.0511 (18)	0.028 (2)	0.000	0.000	−0.0067 (17)
Cl2	0.042 (2)	0.043 (2)	0.0198 (16)	0.000	0.000	0.0094 (13)
N1	0.022 (2)	0.022 (2)	0.022 (2)	0.000	0.000	0.0000 (10)

### Geometric parameters (Å, °)

**Table:**

Hg1—N1	2.192 (8)	C1—H1B	0.9700
Hg1—Cl2	2.311 (3)	C2—N1	1.476 (9)
Hg1—Cl1^i^	2.8083 (17)	C2—C2^ii^	1.549 (15)
Hg1—Cl1	2.8083 (17)	C2—H2A	0.9700
C1—N1	1.490 (13)	C2—H2B	0.9700
C1—C1^ii^	1.52 (2)	Cl1—Hg1^i^	2.8083 (17)
C1—H1A	0.9700	N1—C2^iii^	1.476 (9)
			
N1—Hg1—Cl2	161.7 (3)	N1—C2—H2A	109.6
N1—Hg1—Cl1^i^	94.82 (18)	C2^ii^—C2—H2A	109.6
Cl2—Hg1—Cl1^i^	98.39 (5)	N1—C2—H2B	109.6
N1—Hg1—Cl1	94.82 (18)	C2^ii^—C2—H2B	109.6
Cl2—Hg1—Cl1	98.39 (5)	H2A—C2—H2B	108.2
Cl1^i^—Hg1—Cl1	87.21 (7)	Hg1—Cl1—Hg1^i^	92.79 (7)
N1—C1—C1^ii^	110.4 (5)	C2^iii^—N1—C2	108.1 (8)
N1—C1—H1A	109.6	C2^iii^—N1—C1	109.1 (6)
C1^ii^—C1—H1A	109.6	C2—N1—C1	109.1 (6)
N1—C1—H1B	109.6	C2^iii^—N1—Hg1	110.4 (5)
C1^ii^—C1—H1B	109.6	C2—N1—Hg1	110.4 (5)
H1A—C1—H1B	108.1	C1—N1—Hg1	109.8 (6)
N1—C2—C2^ii^	110.1 (4)		

Symmetry codes: (i) −*x*+1, −*y*, −*z*+1; (ii) *x*, *y*, −*z*+3/2; (iii) −*x*+1, *y*, *z*.

### Hydrogen-bond geometry (Å, °)

**Table:**

*D*—H···*A*	*D*—H	H···*A*	*D*···*A*	*D*—H···*A*
C2—H2A···Cl2^iv^	0.97	2.77	3.708 (8)	163
C2—H2B···Cl2^v^	0.97	2.78	3.678 (8)	154

Symmetry codes: (iv) *x*−1/2, −*y*+1/2, −*z*+1; (v) *x*, −*y*+1, −*z*+1.
